# Evaluation of Jackknife and Bootstrap for Defining Confidence Intervals for Pairwise Agreement Measures

**DOI:** 10.1371/journal.pone.0019539

**Published:** 2011-05-18

**Authors:** Ana Severiano, João A. Carriço, D. Ashley Robinson, Mário Ramirez, Francisco R. Pinto

**Affiliations:** 1 Instituto de Microbiologia, Instituto de Medicina Molecular, Faculdade de Medicina de Lisboa, Lisboa, Portugal; 2 Departamento de Química e Bioquímica, Centro de Química e Bioquímica, Faculdade de Ciências da Universidade de Lisboa, Lisboa, Portugal; 3 Department of Microbiology, University of Mississippi Medical Center, Jackson, Mississippi, United States of America; University of East Piedmont, Italy

## Abstract

Several research fields frequently deal with the analysis of diverse classification results of the same entities. This should imply an objective detection of overlaps and divergences between the formed clusters. The congruence between classifications can be quantified by clustering agreement measures, including pairwise agreement measures. Several measures have been proposed and the importance of obtaining confidence intervals for the point estimate in the comparison of these measures has been highlighted. A broad range of methods can be used for the estimation of confidence intervals. However, evidence is lacking about what are the appropriate methods for the calculation of confidence intervals for most clustering agreement measures. Here we evaluate the resampling techniques of bootstrap and jackknife for the calculation of the confidence intervals for clustering agreement measures. Contrary to what has been shown for some statistics, simulations showed that the jackknife performs better than the bootstrap at accurately estimating confidence intervals for pairwise agreement measures, especially when the agreement between partitions is low. The coverage of the jackknife confidence interval is robust to changes in cluster number and cluster size distribution.

## Introduction

Biological information is commonly used to cluster or classify entities of interest such as genes, species or samples. Examples are the clustering of gene expression profiles in microarray analysis [1], the grouping of bacterial isolates based on typing methods in the epidemiology of infectious diseases [2], [3] and the tissue distribution pattern of proteins in proteomic analysis [4]. However, different methodologies can be used to cluster the same set of entities, leading to the need for methods that allow the comparison of two clusterings or that determine how well a given clustering agrees with another, especially in the absence of a universally accepted “gold standard” classification [2].

Moreover, facing two different data sources that characterize the same set of biological entities and produce two different clusterings, one may wish to know to what extent, and under which conditions, agreement or disagreement between two clusterings can be maximized. This information may be useful to decide if it is worthwhile to collect and analyze both data sources. If their results are in complete agreement, then it may be enough to collect data from a single source. On the other hand, if the two clusterings disagree, combining their results may offer additional information and discriminatory power. Additionally, if the two data sources carry independent information, clusters that have a good match in both clusterings can be more reliable than clusters resulting from each of the data sources alone [2].

### Clustering agreement measures

The need to compare clusterings has been addressed in such diverse fields as bioinformatics, computer science, psychology and ecology. As a result, different measures have been used and there is no general consensus on the choice of the measure to compare clusterings [2], [5]. A frequent strategy is based upon counting the pairs of entities on which two clusterings agree or disagree. The indices in this class are often known as pairwise agreement measures, and a recent review lists 28 different pairwise agreement measures [5]. However, after correction for chance agreement, many of those measures become equivalent [5]. Although many global measures exist that summarize pairwise comparisons, the adjusted Rand index (AR) remains the most well known and widely used. Some methods provide a global measurement of concordance between clusterings, that also takes into account inter-cluster distances, such as ranked adjusted Rand [2], providing a finer global view. Other methods offer an asymmetric view of concordance, in which the agreement of clustering A with clustering B may be different from the agreement of B with A. An example of this type of measure is the Wallace coefficient (W), which has been applied to the analysis of microbial typing data [3], [6]–[8].

### Confidence intervals

The use and interpretation of clustering agreement measures can be improved by the estimation of suitable confidence intervals (CI). Since the measured concordance is dependent on the particular sample taken from the population, there is variability in the point estimates obtained from the samples relative to those of the true population [9]. Since we are interested in estimating a population parameter using a given sample, CIs are necessary to indicate the reliability of our estimate.

An analytical expression for W CI calculation was recently proposed [9]. However, this method was shown to be valid only under some conditions, in particular for W values greater than 0.5, limiting the calculation of CIs to particular situations. Moreover, an analytical expression is not available for the calculation of CIs for other important and widely used measures, such as AR. In these cases, CIs can be estimated through resampling techniques, that involve withdrawing multiple new samples, called resamples, from the data at hand. To investigate various sampling properties, the estimators are calculated from each of the resamples. Although computer intensive, resampling techniques are very easy to implement and their computational demand is no longer an issue for most applications.

The bootstrap is a resampling method, introduced in 1979, used for estimating a distribution, from which various measures of interest can be calculated (e.g. mean, standard error) [10], [11]. The bootstrap approach makes minimal assumptions, other than that the bootstrap distribution accurately reflects the sampling properties of the estimator, and it is available no matter how mathematically complex the estimator may be. Several variations for calculating bootstrap CIs have been proposed, including the percentile and the bias-corrected and accelerated methods [11]. Additional variations to the bootstrap procedure, mostly used to infer sampling representativeness, have also been applied in the context of ecology [12], [13].

The jackknife is another resampling method allowing for CI estimation. It is frequently seen as a simpler, less computer-intensive version of the bootstrap. The jackknife procedure has been previously applied to calculate CIs for species richness [14], for Simpson's and Shannon's diversity indices [15], and for some pairwise measures, such as Rand [16]. In only a few cases have jackknife and bootstrap methods been directly compared in these contexts [12], [13]. These previous studies have focused on diversity measures and the impact of specific sampling strategies and indicate that sample variability and size determine the most suitable resampling method to be applied, with no clear superiority of jackknife or bootstrap.

### The sampling problem

The main requirement for CI estimation is to know the sampling distribution of the estimator in question [15]. Resampling techniques provide methods to infer sampling distribution properties without assuming a distribution function or knowing analytical expressions for the parameters of the distribution. Applying resampling methods to estimate CIs is a standard procedure [13], [17], [18]. However, depending on the estimator's sampling distribution and on the particular sample available for resampling, the resulting CI may lack the desired properties, namely the probability of containing the population parameter being estimated.

It has been pointed out that many estimators have unsatisfactory sampling properties, especially with small sample sizes [19]. Moreover, it is often not trivial to take a random sample of individuals from a biological population. It was previously emphasized that the theoretical standard errors for diversity indices, in particular, are inappropriate in nearly all cases, because they are derived from the assumption that repeated samples of fixed size are drawn from a homogeneous population, when, in fact, populations are frequently heterogeneous in time and space [14]. These statements are also valid for clustering agreement measures. In fact, these measures can be expected to be extremely sensitive to sampling because of the nature of the measurement itself. Since clustering agreement measures are calculated from the sample and are not an intrinsic property of each sampled entity, small sampling deviations from the population might be amplified by the measurement, as discussed below. These problems may compromise the validity of resampling approaches to estimate CIs for these measures.

Here we evaluate the performance of the most commonly used resampling methods for CI estimation applied to pairwise agreement measures. The evaluation of jackknife in this study was prompted by recent results [20], which indicated that the jackknife might be useful for CI estimation for the adjusted Rand index. To this end, we developed a generally applicable method that compares the CIs of sample estimates with the true parameter of an infinite population. The coverage and average amplitude of the CIs estimated by the bootstrap and the jackknife were evaluated for several pairwise agreement measures: Wallace, Rand and adjusted Rand, Fowlkes & Mallows, Mirkin and Jaccard indices.

## Methods

### Clustering and contingency tables

**Figure 1:**
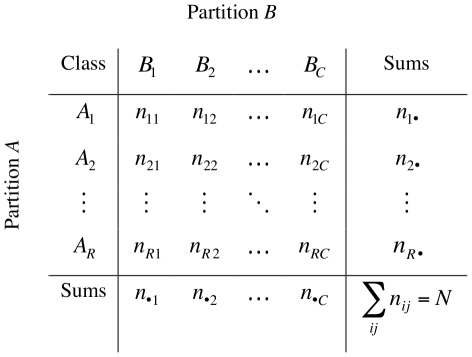
Contingency table (*CT*). *n_ij_* denotes the number of objects that are common to clusters *A_i_* and *B_j_*.

**Figure 2:**
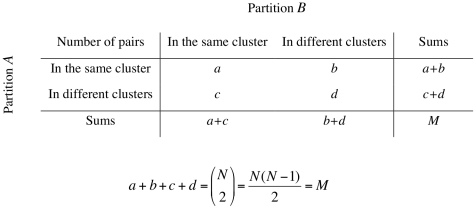
Mismatch Matrix. *a*, *b*, *c* and *d* represent counts of unique entity pairs.

Let *X* be a set of *N* data points {*x*_1_, *x*_2_, *x*_3_, *…*, *x_N_*}. Given two clusterings of *X*, namely *A* = {*A*_1_, *A*_2_, *A*_3_, *…*, *A_R_*} with *R* clusters and *B* = {*B*_1_, *B*_2_, *B*_3_, *…*, *B_C_*} with *C* clusters, the information on cluster overlap between *A* and *B* can be summarized in the form of a *R*×*C* contingency table (CT) as illustrated in figure 1. Every element of *X* contributes to the cell of the corresponding clusters in both *A* and *B*. Focusing on the pairwise agreement, the information in the CT can be further condensed in a mismatch matrix (figure 2). Explicit formulae for calculating *a*, *b*, *c* and *d* in the mismatch matrix can be constructed using entries in the CT [21].

### Construction of the population tables

**Figure 3:**
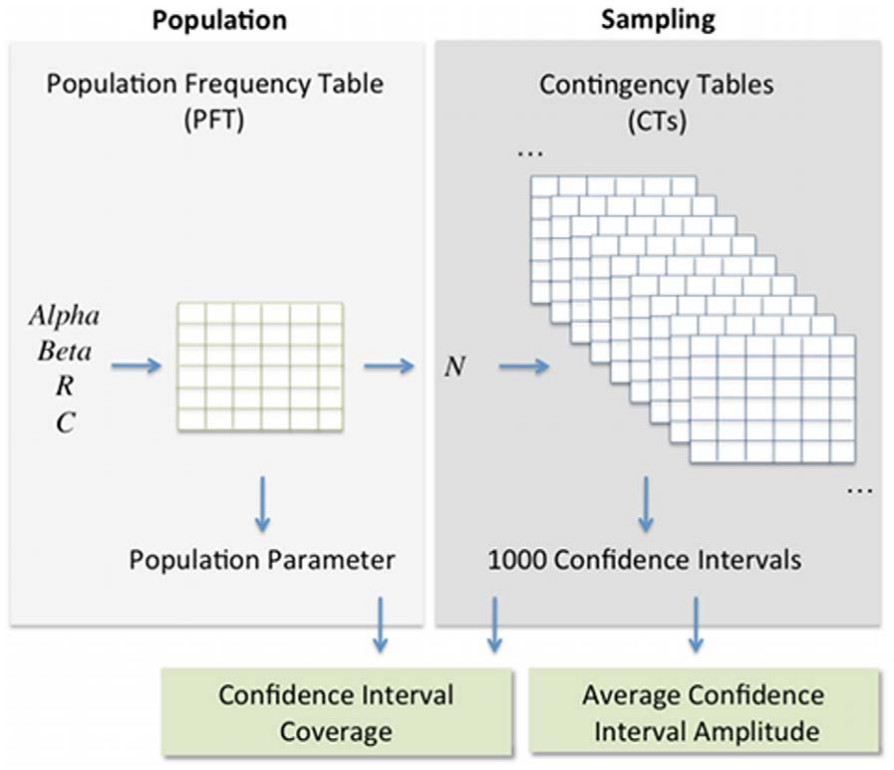
Method used to calculate the coverage and average amplitude of the confidence intervals. The parameters *R*, *C*, *alpha* and *beta* are used to generate a PFT, determining the number of rows, columns and the distribution of cluster size along rows and columns. The population parameter is calculated from the PFT. The sampling process is simulated generating 1000 CTs with *N* elements. The confidence interval is calculated for each one of the CTs. Finally, the coverage is calculated as the fraction of confidence intervals including the population estimate. An average amplitude of the 1000 CIs is also calculated.

In order to simulate the sampling process, population frequency tables (PFT) with *R* rows and *C* columns were randomly generated (figure 3). The total sum of a PFT equals one, representing the CT of an infinite population. The PFTs were generated according to the parameters *R* (number of rows), *C* (number of columns), *alpha* (parameter determining the distribution of cluster sizes in the rows) and *beta* (parameter determining the distribution of the elements in each row across columns). Briefly, the *R* cluster sizes obtained with clustering method *A* were generated according to a Zipfian distribution with exponent *alpha*. This means that ranking clusters by decreasing size, the number of elements in the cluster with rank *z* is proportional to *z^−alpha^*. Then, for each row, a column cluster was randomly selected and the row elements were allocated such that the probability of being assigned to the chosen column cluster is *beta*, and the probability of being assigned to any other cluster is (1−*beta*)/(*C*−1). *Alpha* took the values 0, 0.5, 1, 2 and 3 and *beta* was varied from 0 to 1, with fixed increments of 0.04. Since there is an independent random choice of the column cluster to which elements are assigned with probability *beta* for each row, the overall agreement of a set of PFTs created with the same *alpha* and *beta* parameters can vary substantially. In this way the values of *alpha* and *beta* are not deterministically dictating the overall agreement.

The true population values of Wallace (W), Rand index (RI), adjusted Rand (AR), Jaccard (Jac), Mirkin and Fowlkes & Mallows (FM) indices for each PFT were calculated according to the formulas presented in table 1. All similarity indices listed are function of *a*, *b*, *c*, *d* defined in the mismatch table (figure 2). In the case of an infinite population, the entries of the mismatch table (*a_p_*, *b_p_*, *c_p_* and *b_p_*) are calculated from the PFT entries (*p_ij_*):

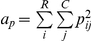

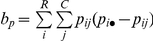

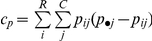

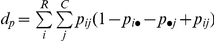

**Table 1:** Pairwise agreement measures.

Measure	Formula	Introduced by
Jaccard	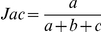	Jaccard (1908) [23]
Rand Index	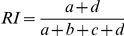	Rand (1971) [24]
Adjusted Rand	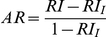 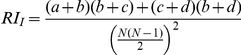	Hubert and Arabie (1985) [21]
Fowlkes and Mallows	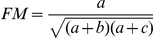	Fowlkes and Mallows (1983) [25]
Wallace coefficient	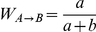	Wallace (1983) [22]
Mirkin metric	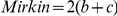	Mirkin (1996) [26]

These expressions define the probabilities of the four possible events when randomly and independently sampling two individuals from an infinite population described by a PFT. Each expression is the sum over all *p_ij_* elements of the product of *p_ij_* itself, corresponding to the first sampled individual, by the sum of PFT entries from which the second individual could be sampled such that it would produce either a cluster match in *A* and *B* (i), in *A* alone (ii), in *B* alone (iii) or a mismatch in both *A* and *B* (iv).

### Simulating sampling from the population

Different sampling processes can be considered depending on the settings where pairwise agreement measures are to be used. If one is interested in comparing two clustering methods on a particular set of individuals to quantify cluster recovery from one method relative to the other, cluster sizes of both clustering methods can be fixed. In this scenario, the CTs may be sampled from a generalized hypergeometric distribution. Another possible scenario is the comparison of the agreement of two methods in classifying individuals from a given population. In this case the set of individuals that is classified can change in each sample. Consequently, the number of partitions and the number of individuals in each partition can vary across samples. If the population is sufficiently large, selection of one individual from the population does not change the probability of sampling a new individual with the same classifications. In other words, this process is equivalent to sampling with replacement and the sampled CTs can be drawn from a multinomial distribution. In a prior publication the latter approach was successfully applied [9]. Additionally, Wallace has argued that even for the first scenario, fixing cluster sizes is not a clearly necessary requirement and may not be even desirable [22]. Both scenarios converge if the number of sampled individuals is large, and, for similar expected frequencies, multivariate hypergeometric distribution presents smaller variances. This difference indicates that CIs that are valid when calculated using the multinomial distribution should also be valid in conditions where the hypergeometric distribution of sampling would be indicated.

Following a multinomial distribution for the absolute frequencies of the PFT, 1000 CTs were randomly generated. Each one of those CTs represents a random sample, of *N* elements, from the infinite population. The CT with *R′* rows and *C′* columns consists in the classifications from two hypothetical clustering methods *A* and *B* for sets of *N* individuals, meaning that method *A* produces *R′* clusters and method *B* produces *C′* clusters. In practice, and in spite of the unbiased way used to generate samples, it is possible (even likely) to miss some cross-classifications that are present in the population in the sampling effort. Therefore, the number of clusters in the population must be regarded as an upper bound of the number of clusters in the sample (*C′*≤*C* and *R′*≤*R*).

For each CT, the 95% CI was estimated by bootstrap and jackknife for each of the pairwise agreement measures being studied. The analytical CI for W was also calculated according to the expression previously derived [9].

### Bootstrap confidence intervals

For each CT, generated by each sample from the population, 1000 independent bootstrap resamples *X* ^*^_1_, *X* ^*^_2_, …, *X* ^*^_1000_ of size *N* were generated. Each bootstrap resample *X* ^*^ = (*x*_1_^*^, *x*_2_^*^, *x*_3_^*^, …, *x_N_* ^*^) was obtained by randomly sampling *N* times, with replacement, from the original data set *X* = *x*_1_, *x*_2_, *x*_3_, …, *x_N_*. To obtain the bootstrap distribution, the pairwise agreement measures were calculated for each of the 1000 bootstrap resamples. The bootstrap indices were then sorted in ascending order.

### Bootstrap percentile method

The bootstrap CI calculated by the percentile method, for an intended coverage of 1−2*α*, is obtained directly from the percentiles *α* and 1−*α* of the bootstrap distribution. Therefore, to obtain the 95% bootstrap percentile CI lower and upper limits, the 25^th^ and 975^th^ values in the ordered bootstrap indices were chosen, since we had 1000 resamples.

### Bootstrap bias-corrected and accelerated method

Efron and Tibshirani [11] proposed a bias-corrected and accelerated method (BC*a*) for calculating CIs. This method adjusts for possible bias in the bootstrap distribution and accounts for the possible change in the standard deviation of an estimator [10]. The CI limits for the BC*a* method, are also given by percentiles in the bootstrap distribution, but those are not necessarily the same ones as in the percentile method.

The percentiles chosen depend on two parameters that can be calculated: the acceleration and the bias-correction. If both numbers equal 0, the BC*a* interval will be the same as the percentile interval. Non-zero values of acceleration and bias-correction will change the percentiles used as the BC*a* endpoints. Therefore, when an estimator is unbiased and its standard deviation does not depend on the true value it is estimating, the BC*a* method will, on average, give the same CI as the percentile method.

### Jackknife confidence intervals

The delete-one jackknife relies on resamples that leave out one entity of the sample at a time, where entities are those individuals that are randomly sampled from the population. Following Smyth et al. [20], a pseudo-values approach was used to calculate the jackknife CIs. For an estimator *S*, the *i* ^th^ pseudo-value of *S* was calculated as
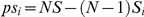
where *S_i_* is the estimator value for the sample with the *i* ^th^ data point deleted. The jackknife CI was then calculated as 
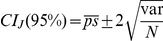
, where 
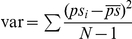
 and 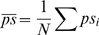.

### CI Coverage and Amplitude

The coverage of a putative CI is the probability that it actually contains the true value. For a 95% CI, the coverage probability should be as close to 0.95 as possible. If the coverage is much higher or lower than 0.95, then the CIs can be misleading.

To calculate the coverage of a CI we consider the contingency table of the population, i.e., the PFT, and not that of individual samples, that may already be biased relative to the population. The CI coverage was calculated as the fraction of the CIs computed from each sample CT that included the value of the pairwise agreement measure computed from the PFT, that constitutes the true population value (see figure 3).

In the present work, each coverage value is computed from the CIs of 1000 samples. As such, each estimate of the coverage, *x*, has a standard error of *s* = (*x*(1−*x*)/1000)^0.5^, and associated 95% CI of *x*±1.96 *s*. This CI of the coverage estimate will have maximum amplitude for a coverage value of 50% (46.9–53.1%), and will decrease for smaller and higher coverage values. For instance, errors associated with the 95% CI for the following coverage estimates are: 80% (77.5–82.5%), 90% (88.1–91.8%), 95% (93.6–96.4%) and 99% (98.4–99.6%).

The amplitude of a CI is defined as the difference between its upper and lower limits. For each population (PFT) the average of the amplitudes calculated for each of the corresponding 1000 samples (CTs) was considered (see figure 3).

## Results and Discussion

**Figure 4:**
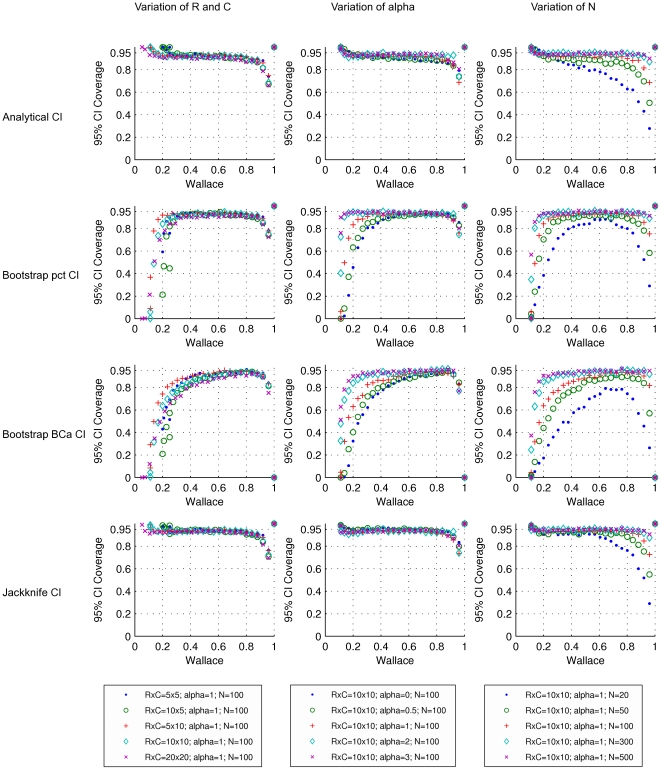
Coverages of 95% confidence intervals for the Wallace coefficient. Rows refer to the methods by which the CIs were calculated. From top to the bottom: analytical formula, bootstrap percentile method, bootstrap BC*a* method and jackknife. Each dot represents a simulated population (PFT), with a particular set of parameters, and 1000 samples from the population (CTs). Symbols and colors represent changes in: dimensions of the simulated probability tables, corresponding to the number of clusters in each of the two classifications (left); exponent *alpha* of the Zipfian distribution determining the distribution of row cluster sizes of the simulated probability tables (middle); sample size or number of elements in the contingency tables (right).

**Figure 5:**
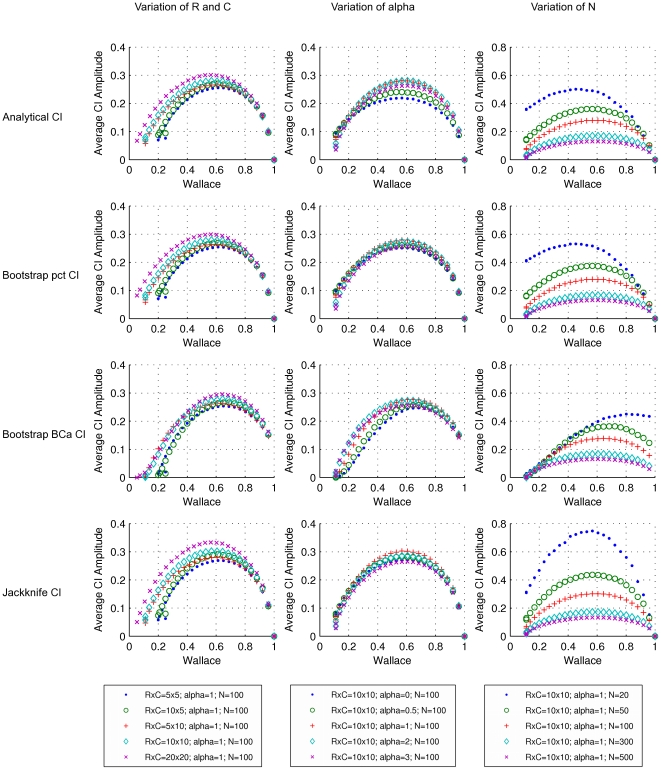
Average amplitudes of 95% confidence intervals for the Wallace coefficient. Rows refer to the methods by which the CIs were calculated. From top to the bottom: analytical formula, bootstrap percentile, bootstrap BC*a* method method and jackknife. Each dot represents a simulated population (PFT), with a particular set of parameters, and the average amplitude of the CIs for 1000 samples from the population (CTs). Symbols and colors represent changes in: dimensions of the simulated probability tables, corresponding to the number of clusters in each of the two classifications (left); exponent *alpha* of the Zipfian distribution determining the distribution of row cluster sizes of the simulated probability tables (middle); sample size or number of elements in the contingency tables (right).

**Figure 6:**
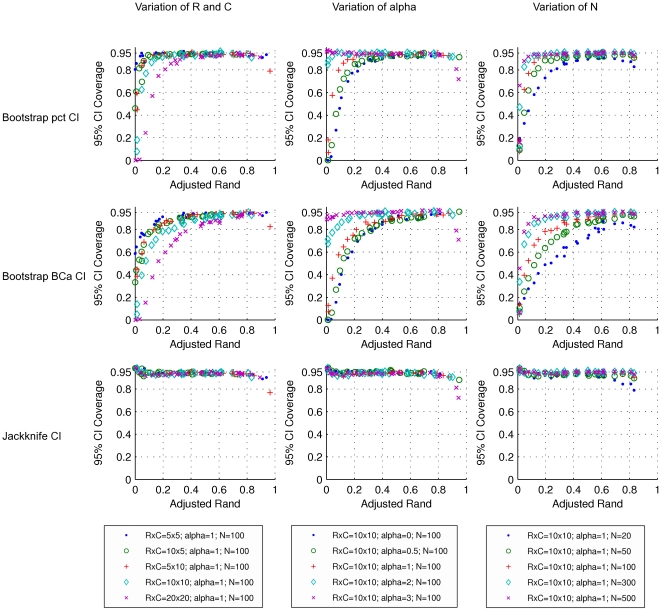
Coverages of 95% confidence intervals for adjusted Rand. Rows refer to the methods by which the CIs were calculated. From top to the bottom: bootstrap percentile method, bootstrap BC*a* method and jackknife. Each dot represents a simulated population (PFT), with a particular set of parameters, and 1000 samples from the population (CTs). Symbols and colors represent changes in: dimensions of the simulated probability tables, corresponding to the number of clusters in each of the two classifications (left); exponent *alpha* of the Zipfian distribution determining the distribution of row cluster sizes of the simulated probability tables (middle); sample size or number of elements in the contingency tables (right).

**Figure 7:**
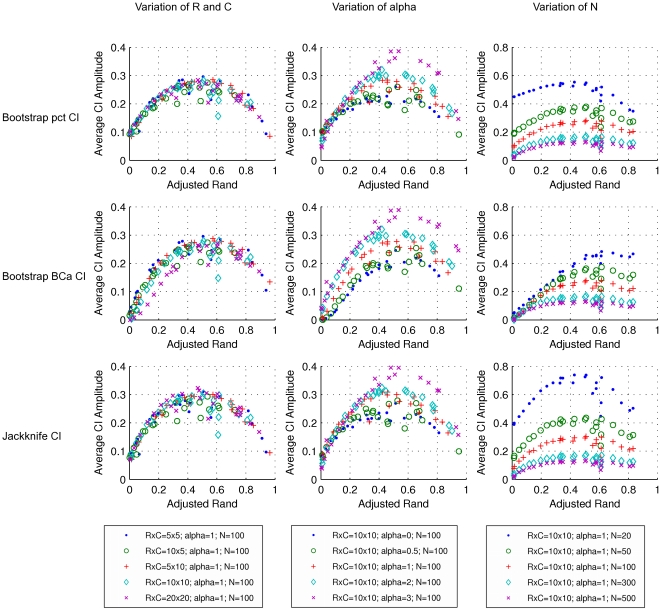
Average amplitudes of 95% confidence intervals for the adjusted Rand. Rows refer to the methods by which the CIs were calculated. From top to the bottom: bootstrap percentile method, bootstrap BC*a* method and jackknife. Each dot represents a simulated population (PFT), with a particular set of parameters, and the average amplitude of the CIs for 1000 samples from the population (CTs). Symbols and colors represent changes in: dimensions of the simulated probability tables, corresponding to the number of clusters in each of the two classifications (left); exponent *alpha* of the Zipfian distribution determining the distribution of row cluster sizes of the simulated probability tables (middle); sample size or number of elements in the contingency tables (right).

The performance of several methods for CI estimation was validated by generating PFTs representing the cross-classification of two hypothetical clusterings in a population and by simulating the sampling process. The results obtained for W and AR are representative of all pairwise agreement measures investigated here and are presented in figures 4, 5, 6, 7. Since there is a known analytical CI for W, we use it as a reference to evaluate how well the resampling CIs perform.

Analysis of the first row in figure 4 indicates that the analytical 95% CI for W approximates the desired coverage of 95% in most of its range (0 to 0.8) for a sample size bigger than 100. This behavior is quite robust to changes in the number of clusters in each of the classifications and to changes in the distribution within clusters. However, for smaller samples of 50 and 20 data points, the 95% CI coverage decreases considerably when W>0.25 and W>0.4 respectively (figure 4, first row, right). Although the analytical CI was calculated as described previously [9], the results shown here differ from the ones previously presented. The difference results from two factors. First, we considered a PFT to perform our study, simulating the sampling process. In the previous study, random contingency tables (rCT) were generated around the sample CT using a multinomial distribution [9]. Secondly, the CI coverage was assessed differently: in the previous study it was calculated as the fraction of W values calculated from rCT that were between the limits of the CI computed from the CT [9]. This corresponds to the interpretation of the CI as a prediction interval and evaluates how well the CI predicts the behaviour of new samples. In the present study we evaluate the probability that the true population value is contained within the CI limits computed for any given sample. The two evaluation strategies are related, and actually agree in a subset of the conditions tested. We believe the methodology in the present work corresponds to a more general interpretation of a CI, and that our results thus complement the ones previously published [9]. Because we considered the PFT, the influence of sample size in CI coverage is more evident, especially when the agreement between clusters is high.

Still considering the analytical CI, we observe that for high values of W (e.g. W>0.8 and *N*>100) the CI coverage gradually decreases, meaning that the 95% CI has in reality a lower coverage and the confidence level of the interval is overestimated (figure 4). This extreme case can be explained by considering the nature of the sampling distribution. When the W of the population approaches its maximum (W = 1), the PFT is very sparse and there is a high probability of missing some of the population's cross-classifications during the sampling effort, resulting in a W value for the sample of 1. When W is 1 for the sample, the CI interval will always be [1,1], which means the amplitude of the interval is zero. Each point in figure 5 represents the average of the amplitudes considering the 1000 CIs calculated. In this figure we can observe that as the W of the population approaches 1, the average amplitude of the analytical CI decreases, reflecting the higher number of zero amplitude CIs. Moreover, unless the W of the population is also 1, the calculated CI will always miss the population value, resulting in a lower coverage. This behavior is more pronounced for smaller samples, because there is an even higher probability of obtaining W = 1.

Considering the coverage for the CI calculated with the bootstrap percentile method (figure 4, second row), there is a decrease in coverage for W>0.8 (*N* = 100), similarly to that observed with the analytical method and previously discussed. In contrast to the analytical CI estimation, the bootstrap percentile method resulted in decreased coverages for lower W values (e.g. when W<0.3 and *N* = 100). Analysis of the bootstrap distributions revealed that in most of these cases the distributions were positively skewed and biased relative to the sample estimate (figure S1). The bootstrap approach is based on the assumption that the bootstrap distribution is similar to the sample distribution. However, the bootstrap process consists of resampling with replacement. When the sample W is low, resampling the same individual several times artificially increases the agreement between partitions, resulting in a biased and skewed bootstrap distribution (figure S1). Because the BC*a* corrects for bias and skewness, we would expect better results with this correction. However, because the bootstrap distribution does not mimic the sample distribution for low W, the BC*a* method resulted in even lower coverages in these cases (figure 4, third row). When BC*a* tries to compensate for the skewness of the distribution, it is in fact dealing with an intrinsic artifact of the resampling method and the types of measures we are using, which does not reflect directly the sampling process. This points to the possibility of biased estimators and suggests that future work should be directed towards identifying better estimators of the population parameter. Comparing the CI of both bootstrap methods, we observe that for small values of W the amplitude of the CI is larger for the percentile method, whereas for high values of W, the amplitude of the CI is larger for the BC*a* method (figure 5, second and third rows). Nevertheless, these differences are only evident for small sample sizes (*N*≤50).

The coverages obtained for the jackknife CI were superior to those of either bootstrap CI. More importantly, jackknife CIs maintained this behavior throughout the whole range of W values and were quite robust to the variation of the parameter tested (see figure 4). In fact, the coverages obtained for the jackknife CI match the performance of the analytical CI and in some cases are marginally better than those obtained analytically (e.g., see the coverages by these two methods for *N* = 20, figure 4, last column). This increase of coverage for the jackknife CI is also reflected in a modest increase in the amplitude of the CI for most parameters tested and that became more pronounced for small values of *N* (*N*≤50) (figure 5, last row). Taken together, these observations suggest that the jackknife provides a viable method to calculate CI for measures for which no analytical formula is known.

According to Efron, “the jackknife uses only limited information about the statistic and thus one might guess that the jackknife is less efficient than the bootstrap” [10]. However, in our study the standard bootstrap resampling procedure was not capable of reproducing the sample distribution for small values of W. When the correction for skewness and bias is applied, we lose even more information about the population, resulting in lower CI coverages (figure 4). So, our results indicate that the jackknife outperforms or matches the bootstrap in the CI estimation of pairwise agreement measures. This is in contrast with previous studies that point to situations where the bootstrap is sometimes superior to the jackknife [11]–[13]. The reasons for this behavior are intrinsic to each procedure and reflect the particular properties of pairwise agreement measures, as discussed above.

As representative of the measures of bi-directional agreement, the results for AR are very similar to the ones observed for W (figures 6 and 7). Again, for *N*>100, the coverage of the jackknife 95% CI is very close to 0.95, independently of the number of clusters and the distribution among clusters. The increase in CI amplitude for small samples noted for W is also apparent for AR (figure 7). The robustness of the jackknife CI indicates that this method should be the method of choice for the estimation of AR CI. Thus, our results confirm and extend those of Smyth et al. [20]. Similar results were obtained for Rand, Mirkin, Jaccard and Fowlkes & Mallows measures (figures S2, S3, S4, S5, S6, S7, S8, S9) indicating that the jackknife is a suitable method to estimate CI for a variety of pairwise agreement measures.

Our study clarifies the sampling and sample size related limitations when resampling techniques are used to estimate CIs of paiwise agreement measures. Simulations exploring the parameter space showed that the jackknife 95% CI has the required coverage for a large range of parameters and pairwise agreement measures. This result is robust to changes in the number of clusters and cluster size distribution. Our data also reinforces the problem of point estimates of concordance measurements based on small sample sizes. As a rule of thumb, and even in ideal sampling conditions, a minimal sample size of *N* = 50 is needed to obtain an acceptable estimate of the population parameter. It is important to note that even with *N* = 50, the CI coverage drops below 95% for W>0.8, which is an unwanted outcome. Overall, the jackknife method is a simple and suitable way to estimate CIs for some widely used pairwise agreement measures in the biological sciences.

## Supporting Information

Figure S1 **Distributions of bootstrap resamples.** Each plot refers to a different population with a Wallace coefficient calculated from a 10×10 PFT (W, red). In each plot, the Wallace for a sample of 100 individuals is shown is blue (W_Sample_). Only one sample from each population is represented. The histogram shows the bootstrap distribution for this sample (1000 resamples). Confidence intervals calculated by the percentile and BC*a* methods are shown in yellow and green.
(TIFF)

Figure S2 **Coverages of 95% confidence intervals for the Rand index.** Rows refer to the methods by which the CIs were calculated. From top to the bottom: bootstrap percentile method, bootstrap BC*a* method and jackknife. Each dot represents a simulated population (PFT), with a particular set of parameters, and 1000 samples from the population (CTs). Symbols and colors represent changes in: dimensions of the simulated probability tables, corresponding to the number of clusters in each of the two classifications (left); exponent *alpha* of the Zipfian distribution determining the distribution of row cluster sizes of the simulated probability tables (middle); sample size or number of elements in the contingency tables (right).
(TIFF)

Figure S3 **Average amplitudes of 95% confidence intervals for the Rand index.** Rows refer to the methods by which the CIs were calculated. From top to the bottom: bootstrap percentile method, bootstrap BC*a* method and jackknife. Each dot represents a simulated population (PFT), with a particular set of parameters, and the average amplitude of the CIs for 1000 samples from the population (CTs). Symbols and colors represent changes in: dimensions of the simulated probability tables, corresponding to the number of clusters in each of the two classifications (left); exponent *alpha* of the Zipfian distribution determining the distribution of row cluster sizes of the simulated probability tables (middle); sample size or number of elements in the contingency tables (right).
(TIFF)

Figure S4 **Coverages of 95% confidence intervals for the Fowlkes & Mallows.** Rows refer to the methods by which the CIs were calculated. From top to the bottom: bootstrap percentile method, bootstrap BC*a* method and jackknife. Each dot represents a simulated population (PFT), with a particular set of parameters, and 1000 samples from the population (CTs). Symbols and colors represent changes in: dimensions of the simulated probability tables, corresponding to the number of clusters in each of the two classifications (left); exponent *alpha* of the Zipfian distribution determining the distribution of row cluster sizes of the simulated probability tables (middle); sample size or number of elements in the contingency tables (right).
(TIFF)

Figure S5 **Average amplitudes of 95% confidence intervals for the Fowlkes & Mallows.** Rows refer to the methods by which the CIs were calculated. From top to the bottom: bootstrap percentile method, bootstrap BC*a* method and jackknife. Each dot represents a simulated population (PFT), with a particular set of parameters, and the average amplitude of the CIs for 1000 samples from the population (CTs). Symbols and colors represent changes in: dimensions of the simulated probability tables, corresponding to the number of clusters in each of the two classifications (left); exponent *alpha* of the Zipfian distribution determining the distribution of row cluster sizes of the simulated probability tables (middle); sample size or number of elements in the contingency tables (right).
(TIFF)

Figure S6 **Coverages of 95% confidence intervals for the Jaccard metric.** Rows refer to the methods by which the CIs were calculated. From top to the bottom: bootstrap percentile method, bootstrap BC*a* method and jackknife. Each dot represents a simulated population (PFT), with a particular set of parameters, and 1000 samples from the population (CTs). Symbols and colors represent changes in: dimensions of the simulated probability tables, corresponding to the number of clusters in each of the two classifications (left); exponent *alpha* of the Zipfian distribution determining the distribution of row cluster sizes of the simulated probability tables (middle); sample size or number of elements in the contingency tables (right).
(TIFF)

Figure S7 **Average amplitudes of 95% confidence intervals for the Jaccard metric.** Rows refer to the methods by which the CIs were calculated. From top to the bottom: bootstrap percentile method, bootstrap BC*a* method and jackknife. Each dot represents a simulated population (PFT), with a particular set of parameters, and the average amplitude of the CIs for 1000 samples from the population (CTs). Symbols and colors represent changes in: dimensions of the simulated probability tables, corresponding to the number of clusters in each of the two classifications (left); exponent *alpha* of the Zipfian distribution determining the distribution of row cluster sizes of the simulated probability tables (middle); sample size or number of elements in the contingency tables (right).
(TIFF)

Figure S8 **Coverages of 95% confidence intervals for the Mirkin metric.** Rows refer to the methods by which the CIs were calculated. From top to the bottom: bootstrap percentile method, bootstrap BC*a* method and jackknife. Each dot represents a simulated population (PFT), with a particular set of parameters, and 1000 samples from the population (CTs). Symbols and colors represent changes in: dimensions of the simulated probability tables, corresponding to the number of clusters in each of the two classifications (left); exponent *alpha* of the Zipfian distribution determining the distribution of row cluster sizes of the simulated probability tables (middle); sample size or number of elements in the contingency tables (right).
(TIFF)

Figure S9 **Average amplitudes of 95% confidence intervals for the Mirkin metric.** Rows refer to the methods by which the CIs were calculated. From top to the bottom: bootstrap percentile method, bootstrap BC*a* method and jackknife. Each dot represents a simulated population (PFT), with a particular set of parameters, and the average amplitude of the CIs for 1000 samples from the population (CTs). Symbols and colors represent changes in: dimensions of the simulated probability tables, corresponding to the number of clusters in each of the two classifications (left); exponent *alpha* of the Zipfian distribution determining the distribution of row cluster sizes of the simulated probability tables (middle); sample size or number of elements in the contingency tables (right).
(TIFF)
