# Supplementary figures and images for: Evaluation of Jackknife and Bootstrap for Defining Confidence Intervals for Pairwise Agreement Measures
Source: PLoS One. 2011 May 18;6(5):e19539. doi: 10.1371/journal.pone.0019539 (PMC3097183; doi:10.1371/journal.pone.0019539)

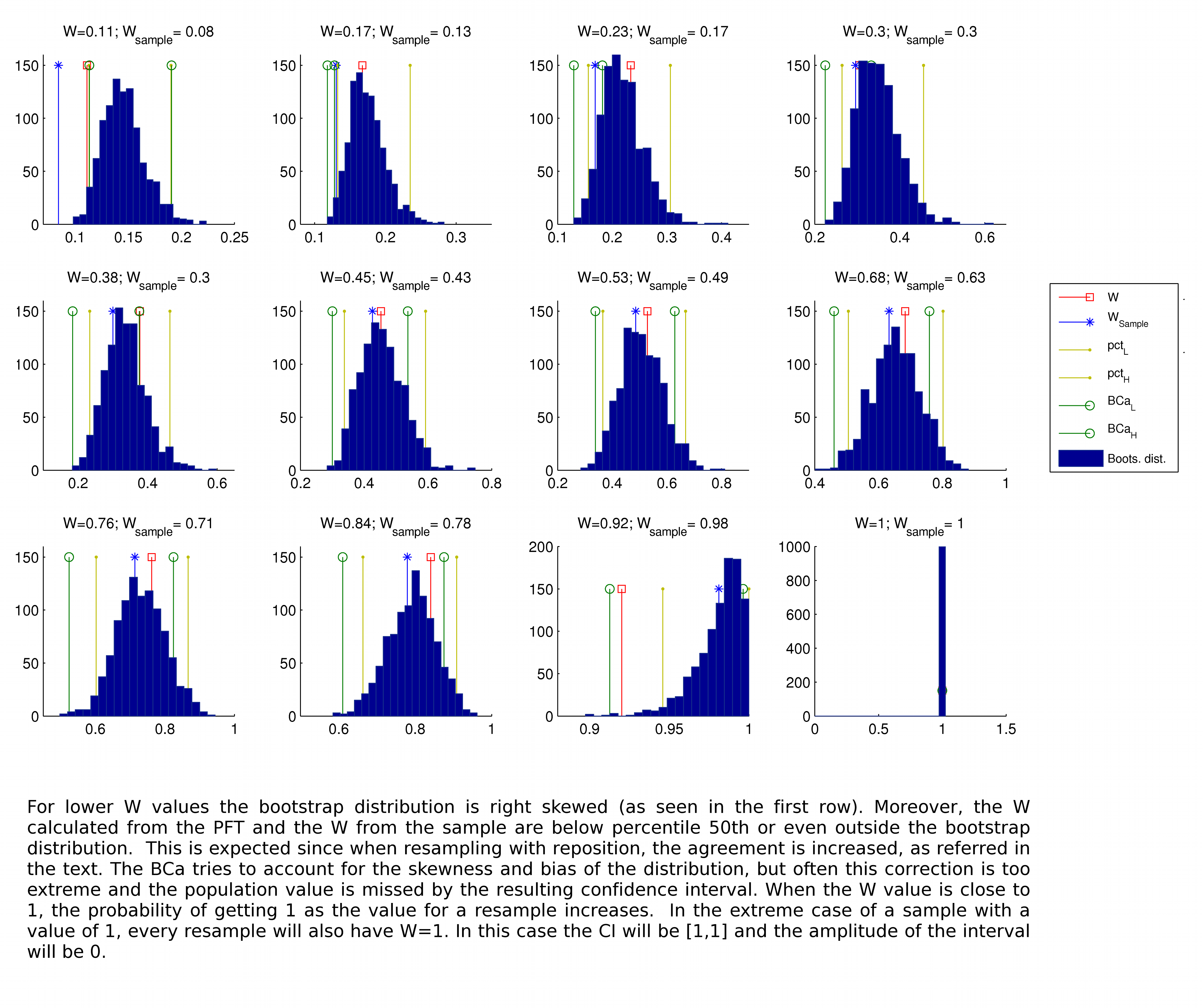

Supplement: Figure S1 — Distributions of bootstrap resamples. Each plot refers to a different population with a Wallace coefficient calculated from a 10×10 PFT (W, red). In each plot, the Wallace for a sample of 100 individuals is shown is blue (WSample). Only one sample from each population is represented. The histogram shows the bootstrap distribution for this sample (1000 resamples). Confidence intervals calculated by the percentile and BCa methods are shown in yellow and green. (TIFF) [file pone.0019539.s001.tiff]

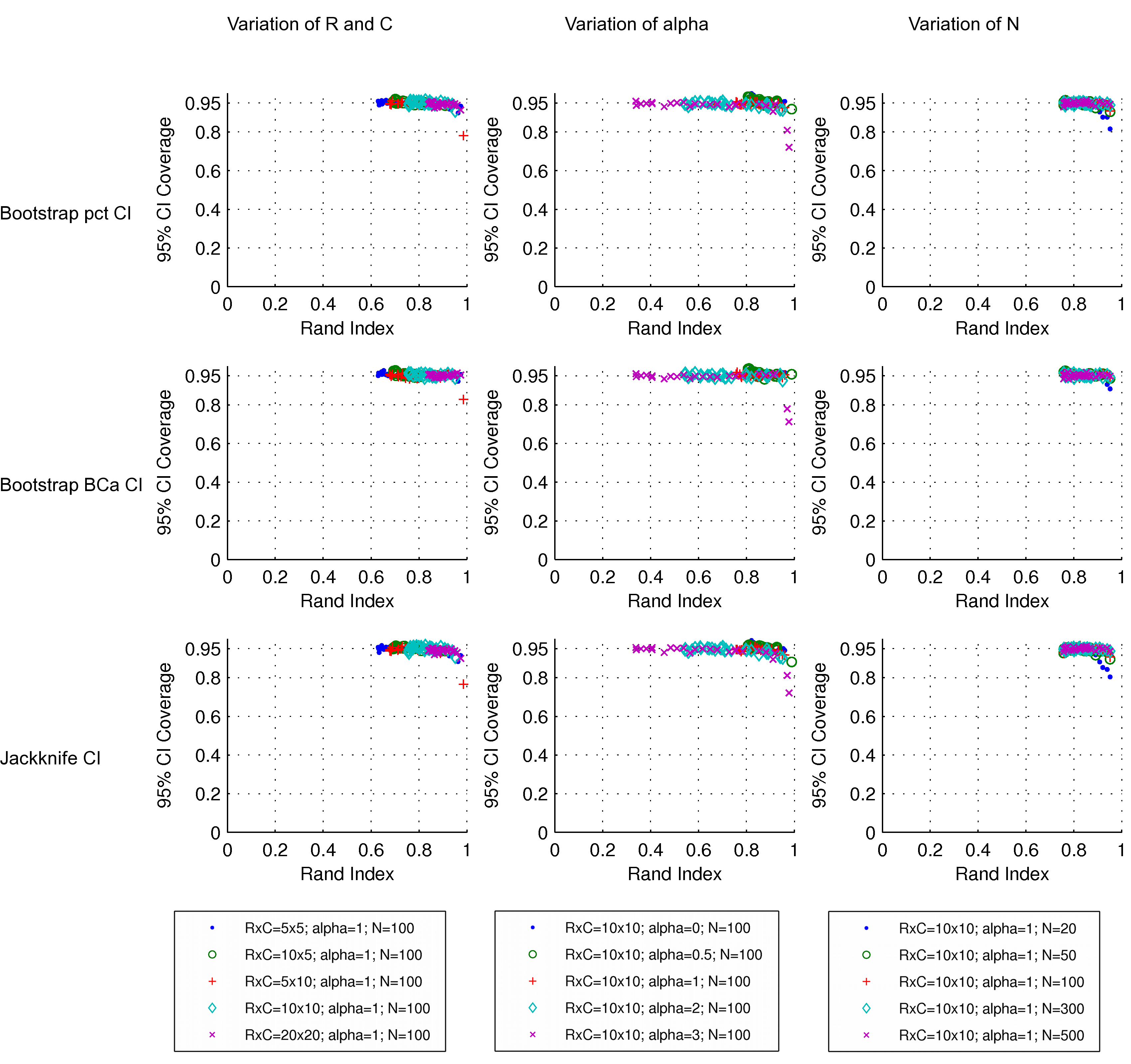

Supplement: Figure S2 — Coverages of 95% confidence intervals for the Rand index. Rows refer to the methods by which the CIs were calculated. From top to the bottom: bootstrap percentile method, bootstrap BCa method and jackknife. Each dot represents a simulated population (PFT), with a particular set of parameters, and 1000 samples from the population (CTs). Symbols and colors represent changes in: dimensions of the simulated probability tables, corresponding to the number of clusters in each of the two classifications (left); exponent alpha of the Zipfian distribution determining the distribution of row cluster sizes of the simulated probability tables (middle); sample size or number of elements in the contingency tables (right). (TIFF) [file pone.0019539.s002.tiff]

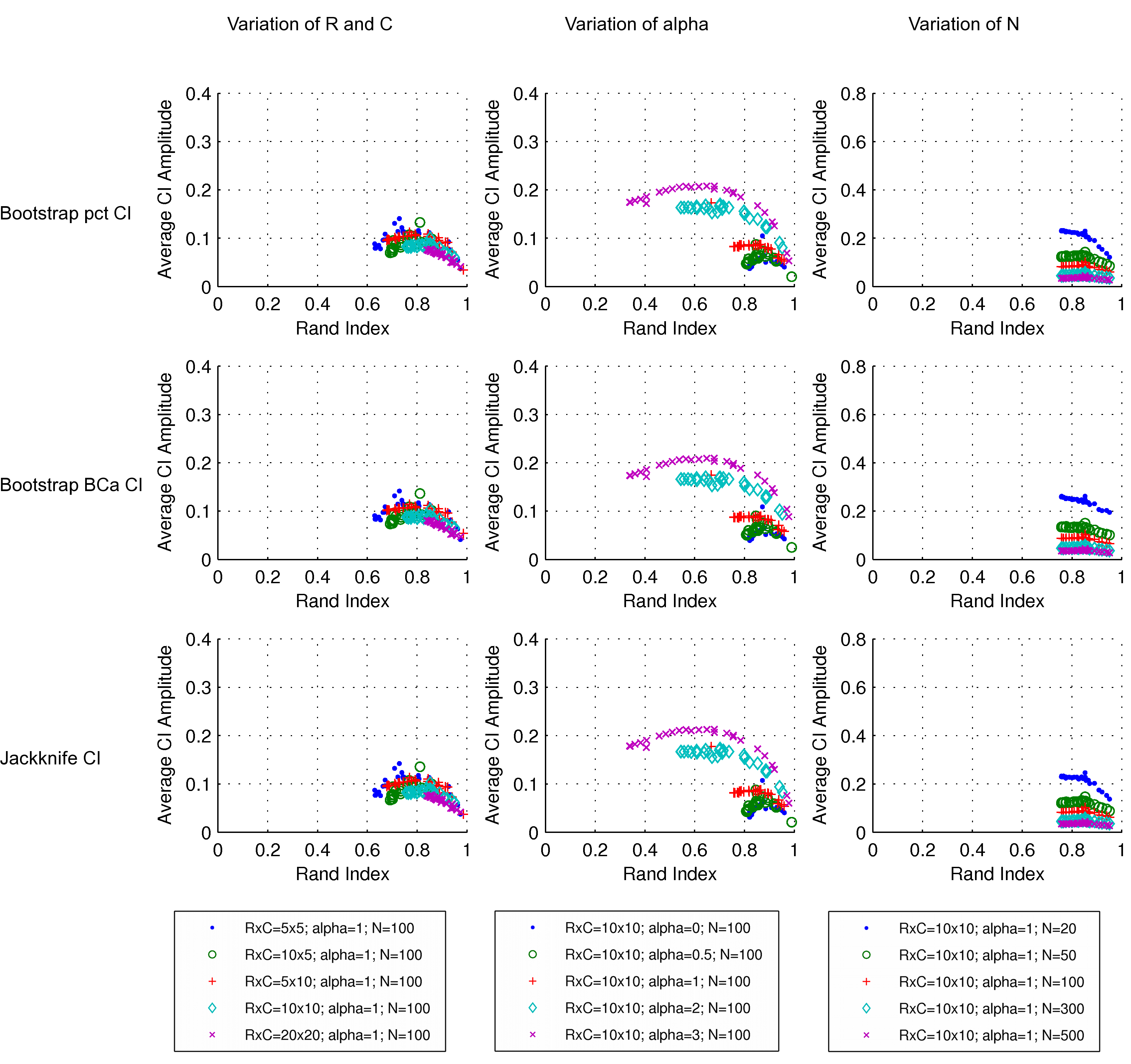

Supplement: Figure S3 — Average amplitudes of 95% confidence intervals for the Rand index. Rows refer to the methods by which the CIs were calculated. From top to the bottom: bootstrap percentile method, bootstrap BCa method and jackknife. Each dot represents a simulated population (PFT), with a particular set of parameters, and the average amplitude of the CIs for 1000 samples from the population (CTs). Symbols and colors represent changes in: dimensions of the simulated probability tables, corresponding to the number of clusters in each of the two classifications (left); exponent alpha of the Zipfian distribution determining the distribution of row cluster sizes of the simulated probability tables (middle); sample size or number of elements in the contingency tables (right). (TIFF) [file pone.0019539.s003.tiff]

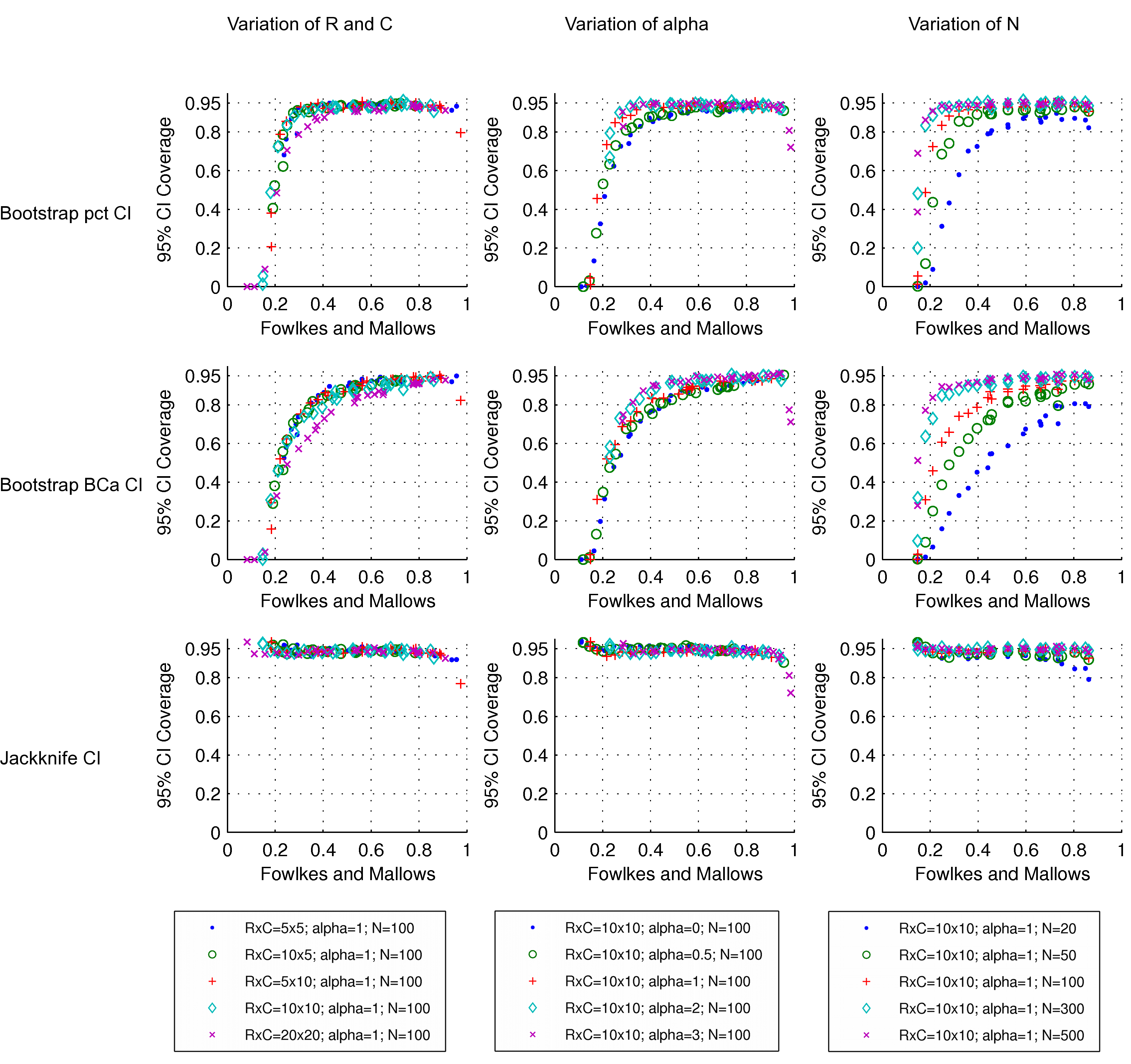

Supplement: Figure S4 — Coverages of 95% confidence intervals for the Fowlkes & Mallows. Rows refer to the methods by which the CIs were calculated. From top to the bottom: bootstrap percentile method, bootstrap BCa method and jackknife. Each dot represents a simulated population (PFT), with a particular set of parameters, and 1000 samples from the population (CTs). Symbols and colors represent changes in: dimensions of the simulated probability tables, corresponding to the number of clusters in each of the two classifications (left); exponent alpha of the Zipfian distribution determining the distribution of row cluster sizes of the simulated probability tables (middle); sample size or number of elements in the contingency tables (right). (TIFF) [file pone.0019539.s004.tiff]

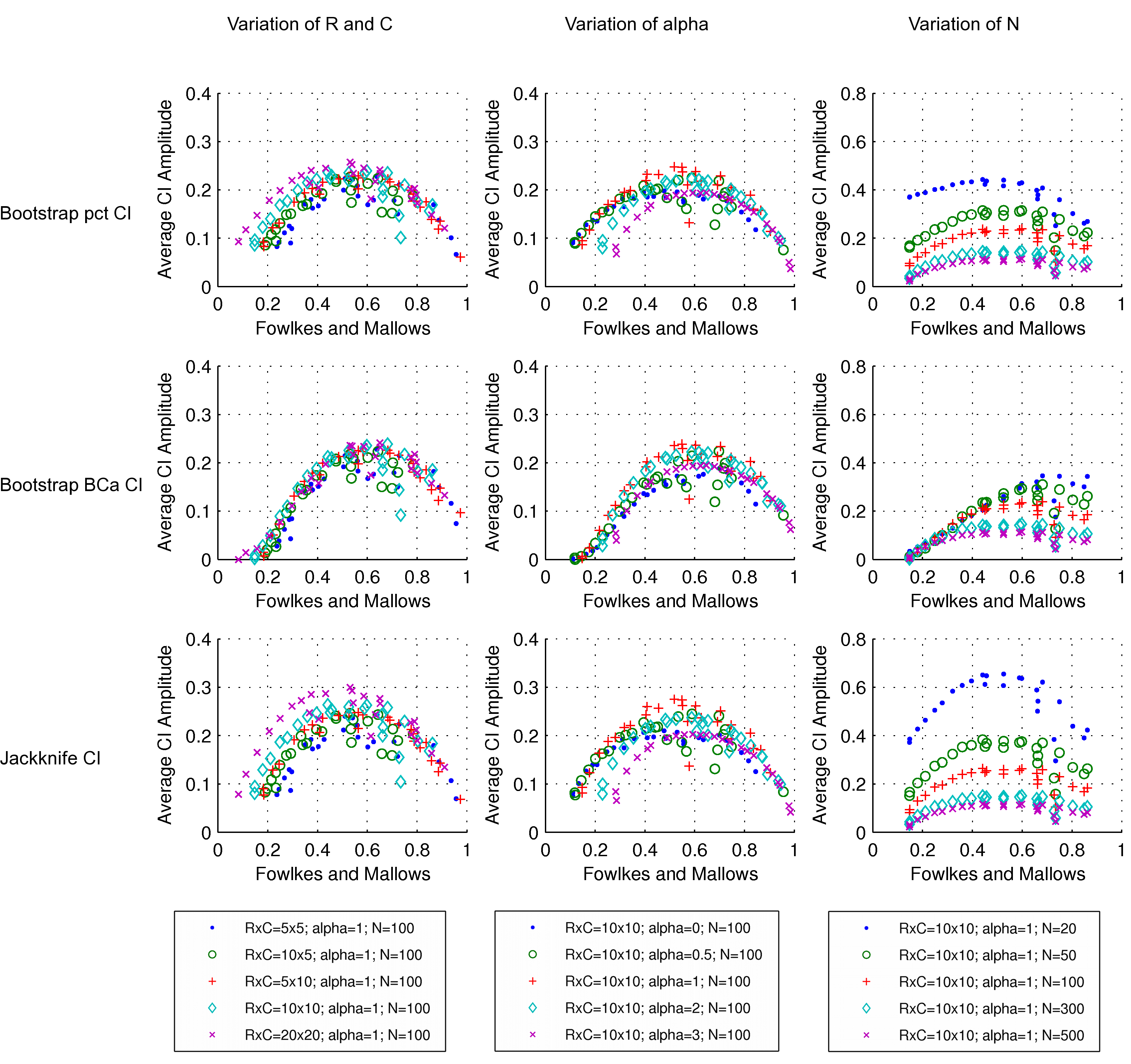

Supplement: Figure S5 — Average amplitudes of 95% confidence intervals for the Fowlkes & Mallows. Rows refer to the methods by which the CIs were calculated. From top to the bottom: bootstrap percentile method, bootstrap BCa method and jackknife. Each dot represents a simulated population (PFT), with a particular set of parameters, and the average amplitude of the CIs for 1000 samples from the population (CTs). Symbols and colors represent changes in: dimensions of the simulated probability tables, corresponding to the number of clusters in each of the two classifications (left); exponent alpha of the Zipfian distribution determining the distribution of row cluster sizes of the simulated probability tables (middle); sample size or number of elements in the contingency tables (right). (TIFF) [file pone.0019539.s005.tiff]

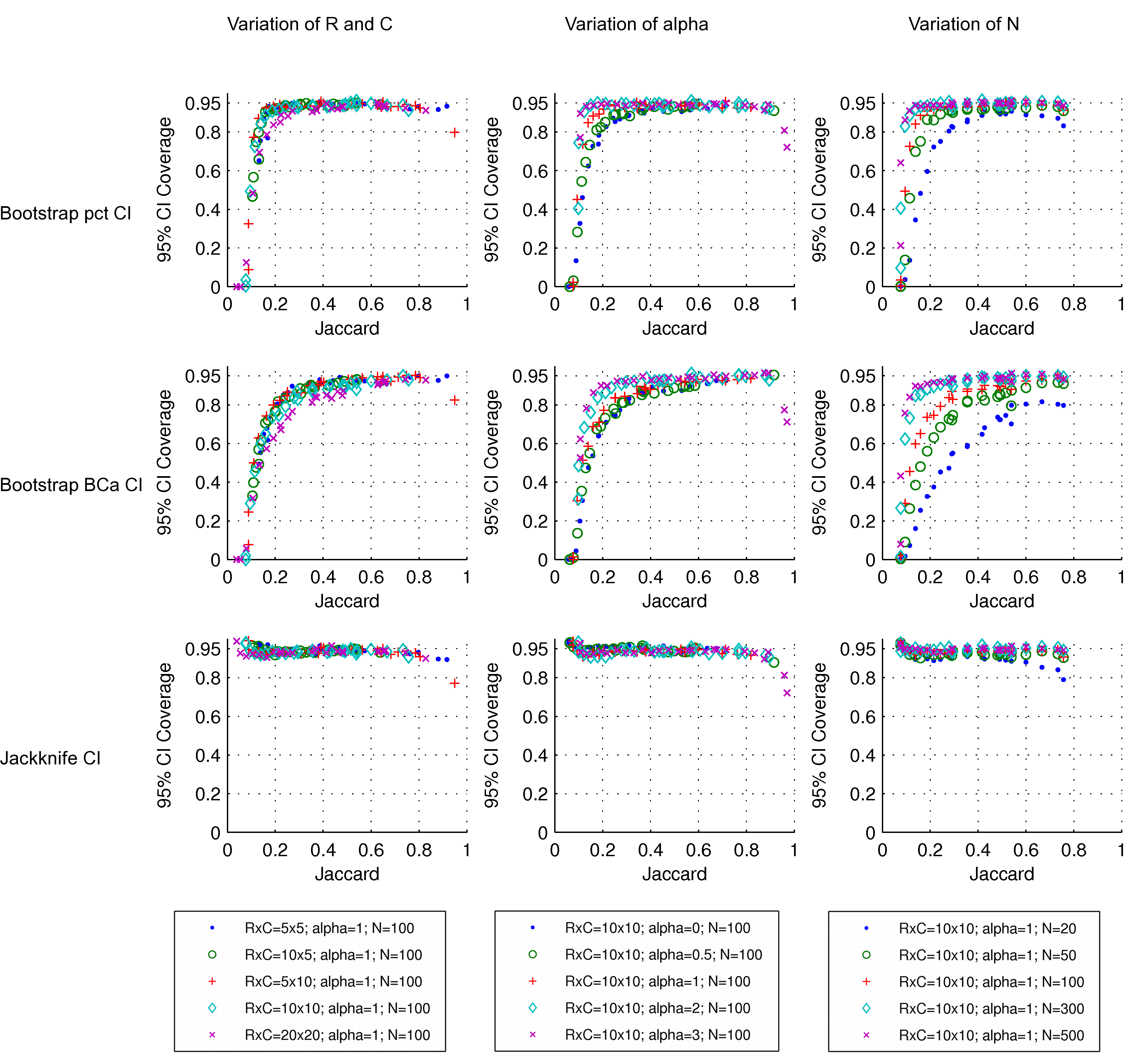

Supplement: Figure S6 — Coverages of 95% confidence intervals for the Jaccard metric. Rows refer to the methods by which the CIs were calculated. From top to the bottom: bootstrap percentile method, bootstrap BCa method and jackknife. Each dot represents a simulated population (PFT), with a particular set of parameters, and 1000 samples from the population (CTs). Symbols and colors represent changes in: dimensions of the simulated probability tables, corresponding to the number of clusters in each of the two classifications (left); exponent alpha of the Zipfian distribution determining the distribution of row cluster sizes of the simulated probability tables (middle); sample size or number of elements in the contingency tables (right). (TIFF) [file pone.0019539.s006.tiff]

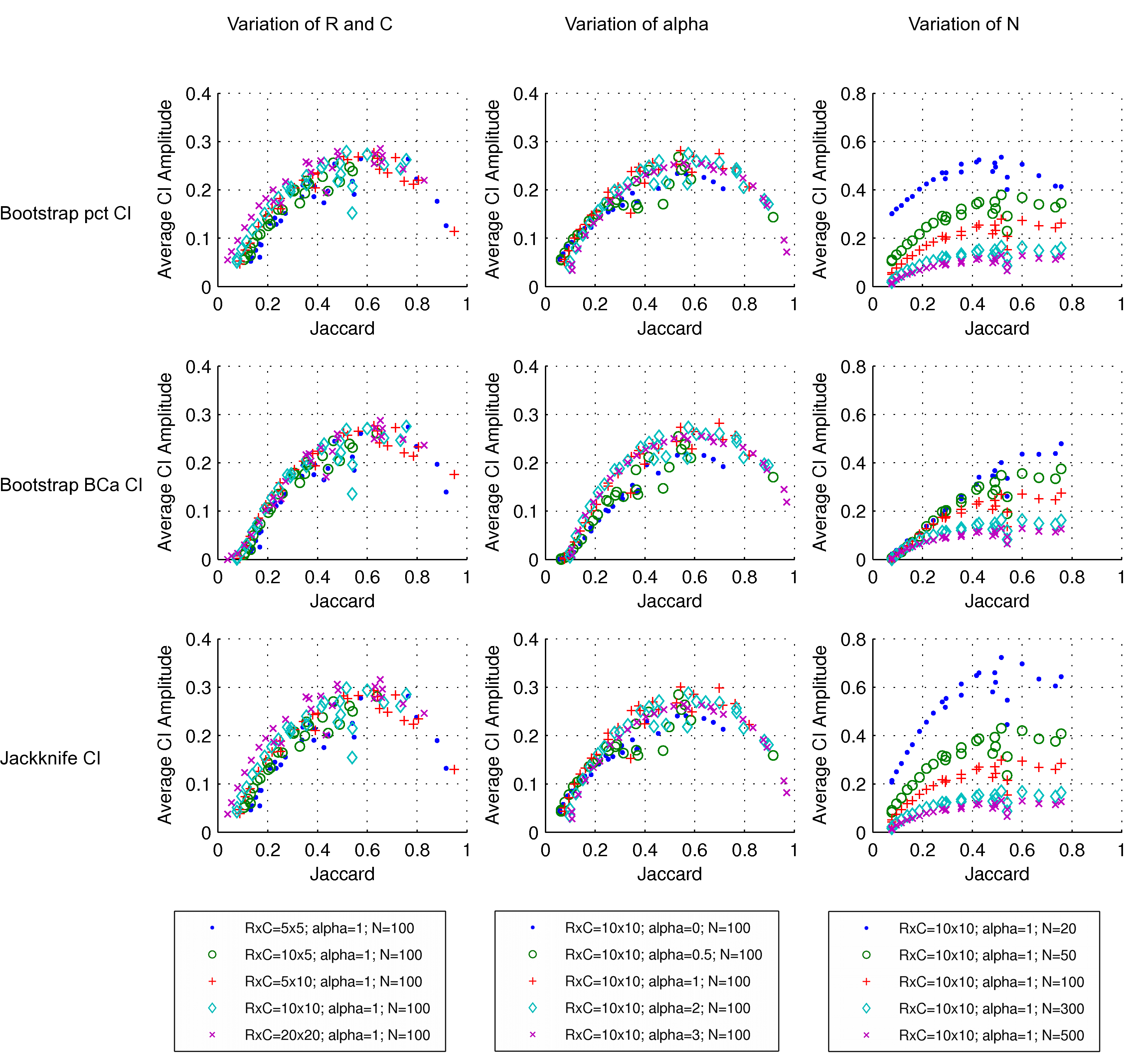

Supplement: Figure S7 — Average amplitudes of 95% confidence intervals for the Jaccard metric. Rows refer to the methods by which the CIs were calculated. From top to the bottom: bootstrap percentile method, bootstrap BCa method and jackknife. Each dot represents a simulated population (PFT), with a particular set of parameters, and the average amplitude of the CIs for 1000 samples from the population (CTs). Symbols and colors represent changes in: dimensions of the simulated probability tables, corresponding to the number of clusters in each of the two classifications (left); exponent alpha of the Zipfian distribution determining the distribution of row cluster sizes of the simulated probability tables (middle); sample size or number of elements in the contingency tables (right). (TIFF) [file pone.0019539.s007.tiff]

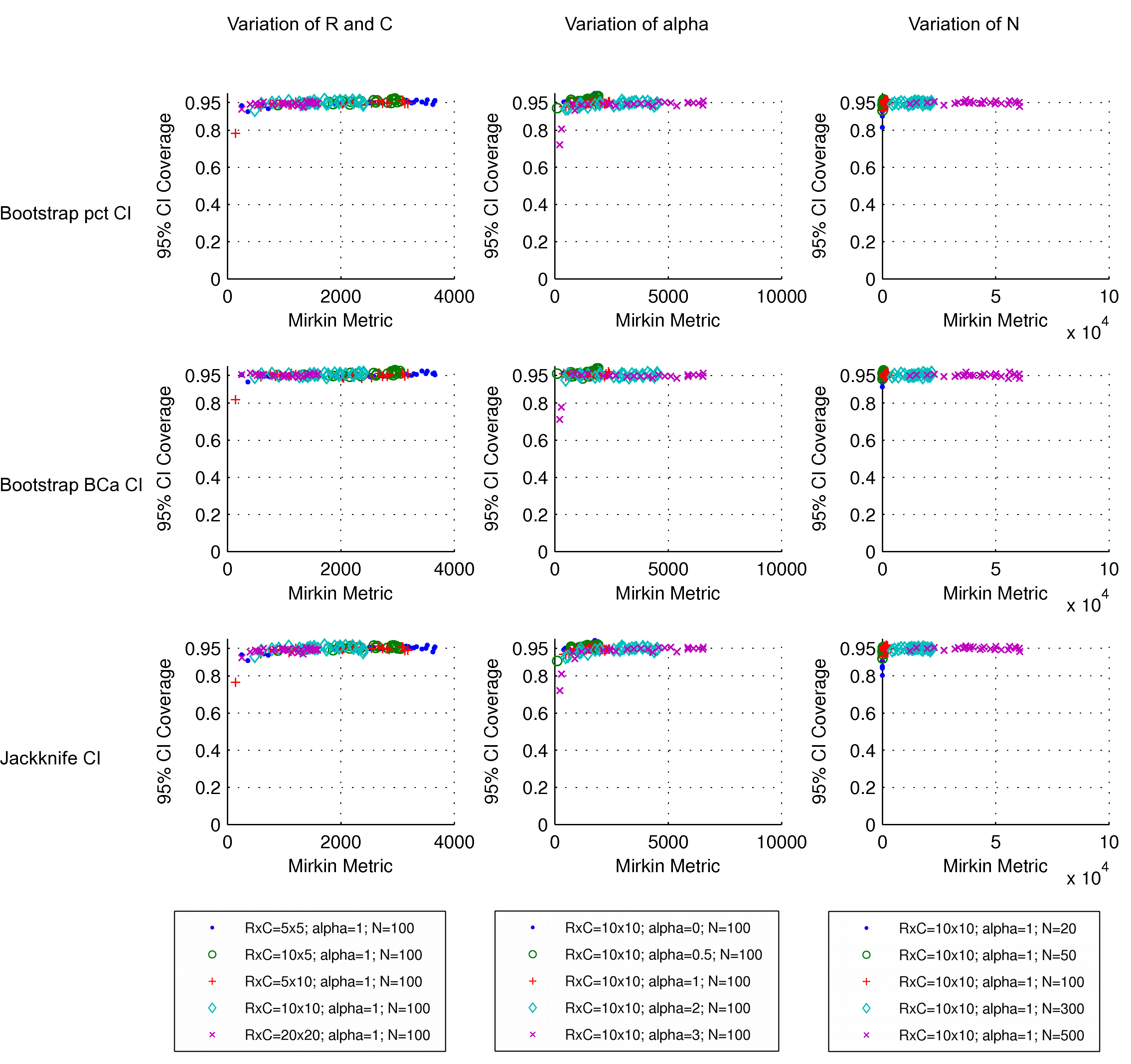

Supplement: Figure S8 — Coverages of 95% confidence intervals for the Mirkin metric. Rows refer to the methods by which the CIs were calculated. From top to the bottom: bootstrap percentile method, bootstrap BCa method and jackknife. Each dot represents a simulated population (PFT), with a particular set of parameters, and 1000 samples from the population (CTs). Symbols and colors represent changes in: dimensions of the simulated probability tables, corresponding to the number of clusters in each of the two classifications (left); exponent alpha of the Zipfian distribution determining the distribution of row cluster sizes of the simulated probability tables (middle); sample size or number of elements in the contingency tables (right). (TIFF) [file pone.0019539.s008.tiff]

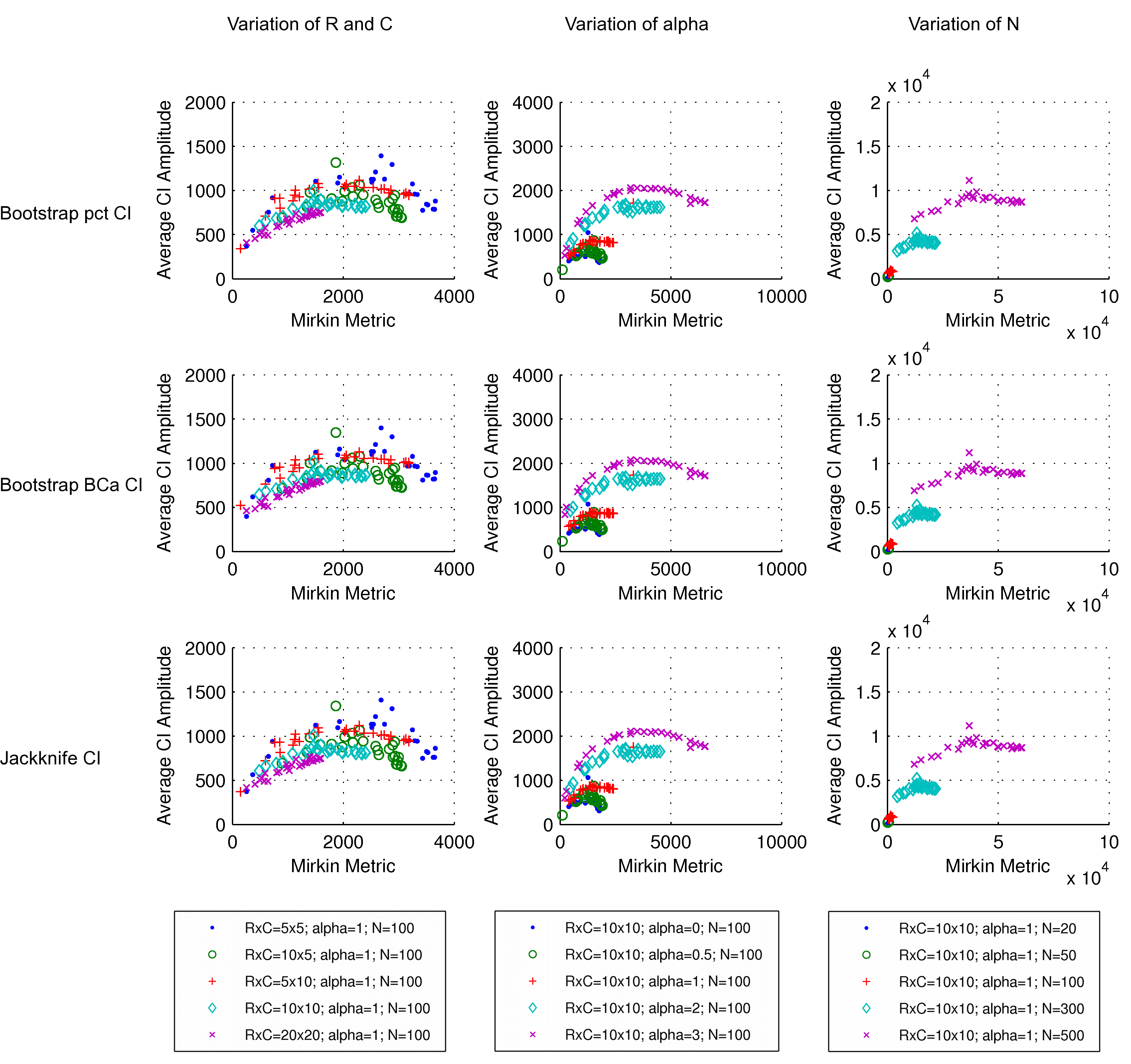

Supplement: Figure S9 — Average amplitudes of 95% confidence intervals for the Mirkin metric. Rows refer to the methods by which the CIs were calculated. From top to the bottom: bootstrap percentile method, bootstrap BCa method and jackknife. Each dot represents a simulated population (PFT), with a particular set of parameters, and the average amplitude of the CIs for 1000 samples from the population (CTs). Symbols and colors represent changes in: dimensions of the simulated probability tables, corresponding to the number of clusters in each of the two classifications (left); exponent alpha of the Zipfian distribution determining the distribution of row cluster sizes of the simulated probability tables (middle); sample size or number of elements in the contingency tables (right). (TIFF) [file pone.0019539.s009.tiff]
